# Intrinsic functional connectivity of blue and red brains: neurobiological evidence of different stress resilience between political attitudes

**DOI:** 10.1038/s41598-020-72980-x

**Published:** 2020-09-28

**Authors:** Taekwan Kim, Ji-Won Hur, Seoyeon Kwak, Dayk Jang, Sang-Hun Lee, Jun Soo Kwon

**Affiliations:** 1 Department of Brain and Cognitive Sciences, Seoul National University College of Natural Sciences, Seoul, Republic of Korea; 2 Department of Psychology, Korea University, Seoul, Republic of Korea; 3 Interdisciplinary Program in Cognitive Science, Seoul National University College of Liberal Studies, Seoul, Republic of Korea; 4 Department of Psychiatry, Seoul National University College of Medicine, Seoul, Republic of Korea; 5 Institute of Human Behavioral Medicine, SNU-MRC, Seoul, Republic of Korea

**Keywords:** Neuroscience, Cognitive neuroscience, Social neuroscience, Stress and resilience

## Abstract

Conservatives are more sensitive to threatening/anxious situations in perceptual and cognitive levels, experiencing emotional responses and stress, while liberals are more responsive to but tolerant of ambiguous and uncertain information. Interestingly, conservatives have greater psychological well-being and are more satisfied with their lives than liberals despite their psychological vulnerability to stress caused by threat and anxiety sensitivities. We investigated whether conservatives have greater resilience and self-regulation capacity, which are suggested to be psychological buffers that enhance psychological well-being, than liberals and moderates. We also explored associations between intrinsic functional brain organization and these psychological resources to expand our neurobiological understanding of self-regulatory processes in neuropolitics. We found that conservatives, compared to liberals and moderates, had greater psychological resilience and self-regulation capacity that were attributable to greater impulse control and causal reasoning. Stronger intrinsic connectivities between the orbitofrontal cortex (OFC) and precuneus and between the insula and frontal pole/OFC in conservatives were correlated with greater resilience and self-regulation capacity. These results suggest the neural underpinnings that may allow conservatives to manage the psychological stress and achieve greater life satisfaction. This study provides neuroscientific evidence for the different responses of liberals and conservatives to politically relevant social issues.

## Introduction

When exposed to politically relevant experiences, individuals make judgments based on the subjective evaluation of the social issues and form a political attitude that motivates their political behaviors^1^. This process is called political socialization and is the process by which individuals shape their own political perspectives that conform to the norms and structures of political and economic institutions where they live^2^. Participating in political activities with one’s own political beliefs and even engaging in daily conversations about political issues often inevitably leads to psychological stress, such as worry and irritability, about threatening or conflicting social issues^3^. Political psychology research has found that psychological responses to stressful events are different in people with left and right political attitudes because the way in which individuals perceive and manage threats or cognitive conflicts influences the formation of their political perspectives^4–6^.

Liberals and conservatives hold opposite political belief systems and have marked differences in psychological responses, especially in the self-regulatory processes dealing with stressful issues^7^. Compared with conservatives, liberals are more responsive to ambiguous and uncertain information, but they are also more tolerant of those stresses^8^. Neuroimaging studies have identified the neurobiological mechanisms underlying the tolerance of ambiguity and uncertainty. When experiencing alterations in habitual response tendencies, they enhance the error-related negativity, which is a neural signal generated from the anterior cingulate cortex (ACC) to process conflict information and trigger compensatory cognitive control^9,10^. Conservatives, by contrast, show different psychological processes to threatening, disgusting, or anxious circumstances, which provoke emotional responses and stress^6,11^. While not physiologically reactive to those stimuli, they are more sensitive in perceptual and cognitive (e.g., attention engagement) levels than liberals^6,11,12^. Neuroimaging studies have shown that conservativism is associated with brain regions involved in emotional sensitivity and the processing of threat and anxiety^13,14^. For example, conservatives activate the amygdala to process the threat and anxiety they experience while making risky decisions^14^.

Despite the psychological predispositions vulnerable to threat/anxiety and stress, conservatives have comparable physiological reactions and rather greater psychological well-being, specifically life satisfaction, than liberals^15–17^. It has been suggested that conservatives have some psychological resources in self-protective mechanisms that allow them to cope with stressful issues and achieve life satisfaction^18^. Based on a tendency to strongly endorse the belief in free will, conservatives have greater self-regulation capacity that may function as a psychological buffer to reduce their stress response to threats and anxiety and improve their life satisfaction^19–21^. In addition to self-regulation capacity, greater psychological well-being in conservatives is also thought to be attributable to psychological resilience, which is a protective factor controlling an agitated internal environment and associated with the regulation capacity^22,23^. These studies suggest that resilience and self-regulation capacity are psychological buffers that help conservatives manage sensitively perceived stress and achieve greater psychological well-being and life satisfaction.

For a decade, neuropolitics research has investigated neural correlates of self-regulatory processes with political attitudes to come to an understanding of the psychological differences between liberals and conservatives^10,13,14^. However, the neural underpinnings of the aforementioned psychological buffers need to be studied to expand our understanding of the mental responses of liberals and conservatives to stressful issues. Because psychological resilience and self-regulation are complex processes, the neural underpinnings may not be fully understood by investigations of limited brain regions under certain task conditions^24^. Intrinsic brain organization analysis is an alternative approach to examine how multiple regions communicate with each other without limiting the analysis to a specified task condition^25^. In addition, intrinsic neural activities correspond to task-related activations associated with executive functions^26^. Thus, intrinsic brain organization analysis may be useful to investigate the neural mechanisms underlying these psychological resources in liberals and conservatives.

The present study was conducted to determine the associations of intrinsic brain organization with resilience and the self-regulation capacity that are thought to be psychological buffers in conservatives. We hypothesized that conservatives have greater psychological resilience and self-regulation capacity than liberals based on their function to help stress management. Neuropsychiatry studies have demonstrated that key brain regions regulating negative emotions and stress are decoupled in psychiatric patients^27–30^. Thus, we tested the hypothesis that neural bases of the greater resilience and regulation capacity in conservatives are stronger intrinsic connections of these regions. We also tested, in an exploratory way, global network metrics of resting-state brain in liberals and conservatives to complement the functional connectivity analysis. We intended to identify the neural mechanisms underlying the psychological resources dealing with stress and expand the current understanding of neuropolitics.

## Results

### Sociodemographic and psychological characteristics

We first demonstrated that homoscedasticity of sociodemographic and psychological variables, except the RQT sociability subtotal score (Fligner–Killeen chi-squared = 3.97, *p* = 0.046). The group comparison results of sociodemographic and psychological are presented in Table 1. Participants were not different in terms of their sociodemographic characteristics, except their political attitude scores. The multivariate analysis of variance (MANOVA) results of the Resilience Quotient Test (RQT) totals showed that there were statistically significant differences in psychological resilience scale scores based on political attitudes (Pillai’s trace *V* = 0.14, *F*(6, 196) = 2.40, *p* = 0.030). We found significant group effects for the RQT total and self-regulation & sociability subtotal scores (*Welch’s F*_*total*_(2, 50.88) = 7.19, *p* = 0.002; *Welch’s F*_*self-regulation*_(2, 44.10) = 6.38, *p* = 0.004; *Welch’s F*_*sociability*_(2, 52.86) = 5.32, *p* = 0.008). In the post hoc results, conservatives had greater resilience and self-regulation (*M* = 205.94, *SD* = 13.61; *M* = 68.63, *SD* = 6.82) than liberals (*M* = 192.24, *SD* = 18.75, *p* = 0.011; *M* = 62.52, *SD* = 7.70, *p* = 0.017) and moderates (*M* = 188.23, *SD* = 24.51, *p* = 0.003; *M* = 61.36, *SD* = 7.71, *p* = 0.004). No significant difference was observed in RQT total and subtotal scores between liberals and moderates.

**Table 1:** Sociodemographic and psychological characteristics of participants.

Variables	Liberals (*N* = 42)	Moderates (*N* = 44)	Conservatives (*N* = 16)	Statistics	
	*N*			*X*^2^ (*df*)	*p*
Sex (male/female)	22/20	20/24	9/7	0.71 (2)	0.702
Handedness (left/right/mixed)	3/37/2	4/37/3	1/13/2	1.25 (4)	0.869

	Mean (*SD*)			*F (df)*	*p*
Age (years)	21.10 (1.74)	21.23 (1.85)	22.00 (1.83)	1.47 (2, 99)	0.234
TIV (L)	1.42 (0.10)	1.38 (0.12)	1.40 (0.10)	0.99 (2, 99)	0.375
Education (years)	15.02 (1.28)	15.23 (1.40)	15.38 (1.20)	0.49 (2, 99)	0.613
Socioeconomic status	2.66 (1.09)	2.68 (0.80)	2.44 (0.81)	0.43 (2, 99)	0.652
RQT^a^					
Total	192.24 (18.75)	188.23 (24.51)	205.94 (13.61)	7.19 (2, 50.88)	0.002
Self-regulation	62.52 (7.70)	61.36 (7.71)	68.63 (6.82)	6.38 (2, 44.10)	0.004
Sociability	66.07 (7.35)	65.91 (9.88)	71.06 (4.99)	5.32 (2, 52.86)	0.008
Positivity	63.64 (9.04)	60.96 (10.48)	66.25 (6.17)	2.84 (2, 51.36)	0.068

TIV: total intracranial volume; RQT: Korean version of the Resilience Quotient Test.

^a^Group comparison by Welch’s ANOVA.

The additional MANOVA analysis of the RQT self-regulation subscales showed significant differences depending on political attitudes (Pillai’s trace *V* = 0.14, *F*(6, 196) = 2.43, *p* = 0.027). We observed group effects for causal reasoning and impulse control subscales of the RQT self-regulation scale (*Welch’s F*_*causal reasoning*_(2, 45.62) = 4.74, *p* = 0.014; *Welch’s F*_*impulse control*_(2, 40.95) = 6.12, *p* = 0.005). Pairwise comparisons showed that conservatives had greater causal reasoning and impulse control (*M* = 24.63, *SD* = 2.47; *M* = 22.31, *SD* = 3.34) than liberals (*M* = 22.62, *SD* = 3.37, *p* = 0.046; *M* = 19.67, *SD* = 2.82, *p* = 0.026) and moderates (*M* = 22.36, *SD* = 2.85, *p* = 0.014; *M* = 18.84, *SD* = 3.48, *p* = 0.004). The RQT self-regulation subscale scores were comparable between liberals and moderates.

### Seed-based resting-state functional connectivity

**Figure 1:**
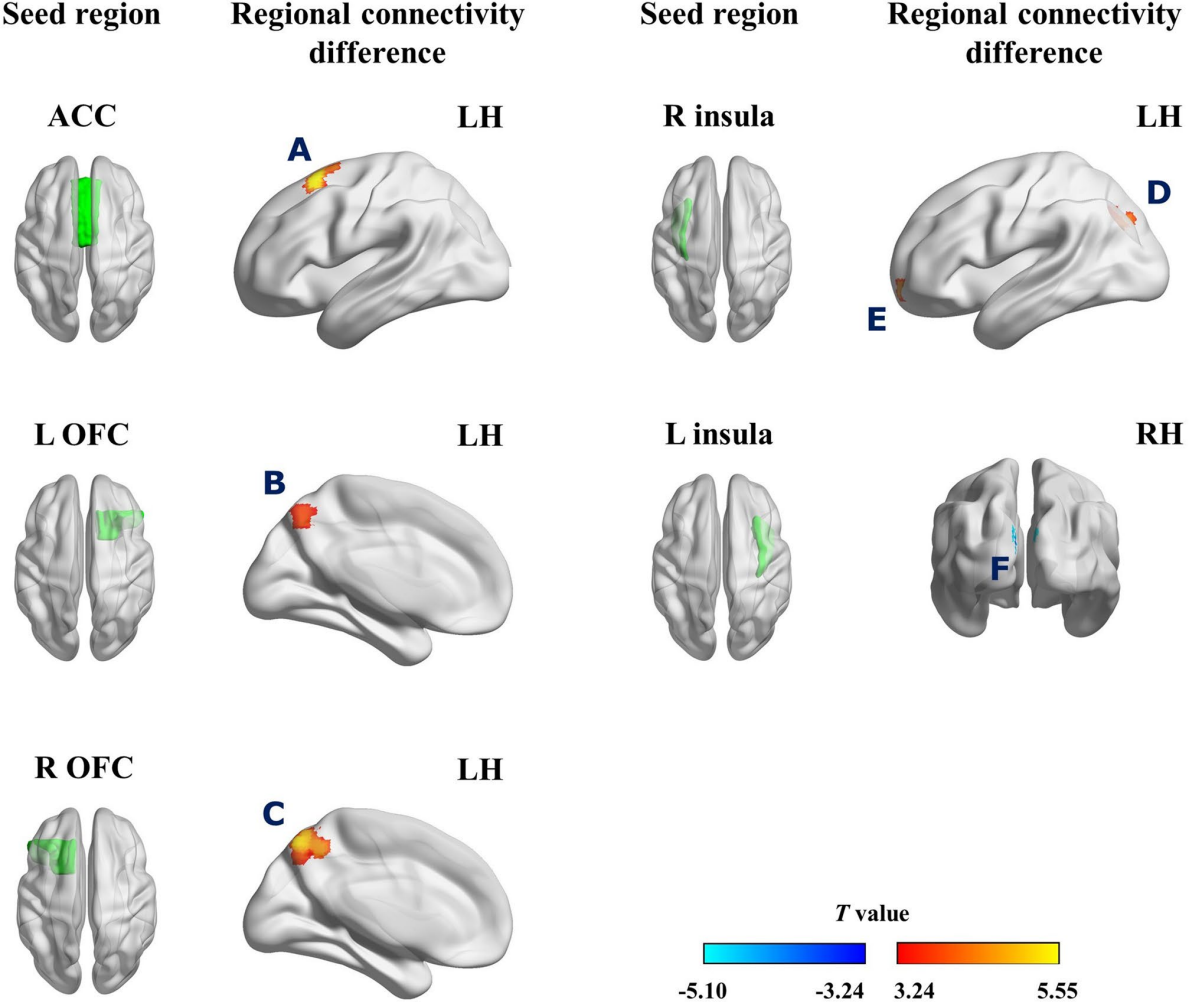
Resting-state functional connectivity differences between liberals and conservatives. Conservatives had stronger anticorrelations of the anterior cingulate cortex (ACC) with (**A**) the superior frontal gyrus (SFG)/dorsolateral prefrontal cortex (dlPFC), of the bilateral orbitofrontal cortex (OFC) with (**B**, **C**) the precuneus, and of the right insula with (**D**) the left superior lateral occipital cortex (LOC) and (**E**) the left frontal pole/OFC, compared to liberals. In addition, (**F**) conservatives had stronger positive correlation between the left insula and the left occipital pole than liberals.

The group comparison results showed that conservatives had stronger anticorrelations between the ACC and the left superior frontal gyrus (SFG)/dorsolateral prefrontal cortex (dlPFC), between the bilateral orbitofrontal cortex (OFC) and the precuneus, and of the right insula with the superior lateral occipital cortex (LOC) and the frontal pole/OFC in the left hemisphere than liberals. In addition, conservatives had a stronger positive correlation of the left insula with the left occipital pole than liberals. There was no difference in resting-state functional connectivities (rsFCs) from the bilateral amygdalae, and all the functional connectivity measures were homoscedastic. We have summarized the detailed results of the seed-based functional connectivity in Table 2 & Fig. 1. The findings of functional connectivities associated with political attitudes were mostly replicated in the regression analysis using one hundred two participants (Table S1).

**Table 2:** Intrinsic functional brain connectivity differences between liberals and conservatives.

Seed region	Brain region	MNI coordinates (mm)	Cluster size (*k*)	Statistics		Connectivity strength (*Z*)	
				*T*	Cluster-extent *p*^a^	Liberals	Conservatives
*Liberals* > *Conservatives*							
ACC	L SFG/dlPFC	−20, 16, 60	131	6.22	< 0.001	0.10	−0.12
L OFC	Precuneus	−12, −60, 46	58	4.86	0.027	−0.05	−0.24
R OFC	Precuneus	−8, −68, 54	274	6.37	< 0.001	0.02	−0.17
R insula	L superior LOC	−26, −72, 32	88	5.04	0.003	−0.01	−0.21
	L frontal pole/OFC	−22, 56, −8	53	4.15	0.026	−0.06	−0.27
*Liberals* < *Conservatives*							
L insula	L occipital pole	−4, −94, 24	74	−4.81	0.009	−0.04	0.17

^a^FDR corrected *p* value.

*ACC* anterior cingulate cortex, *OFC* orbitofrontal cortex, *SFG* superior frontal gyrus, *dlPFC* dorsolateral prefrontal cortex, *LOC* lateral occipital cortex, *L* left; *R* right.

### Global network metrics of functional brain

**Figure 2:**
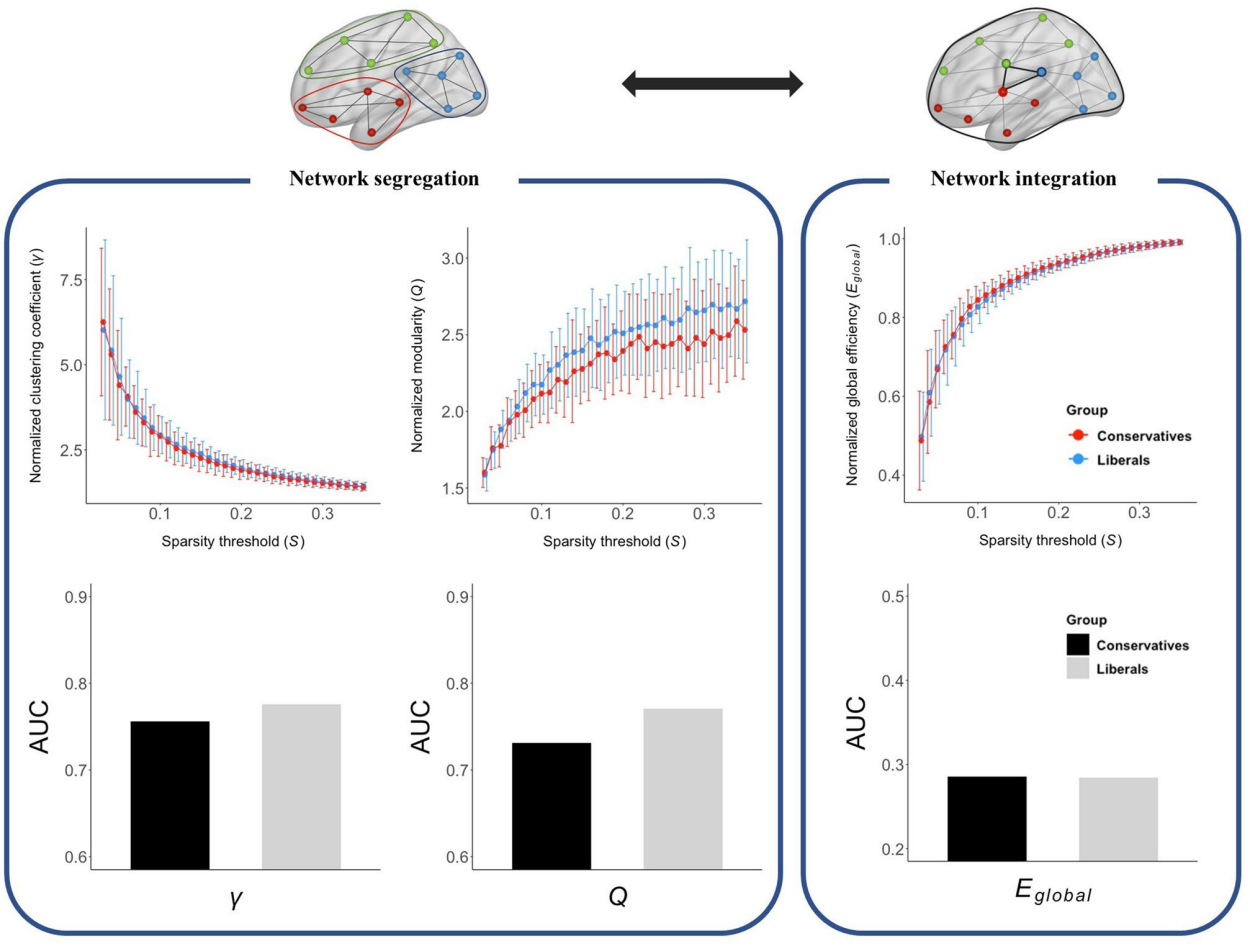
Group comparisons of global network metrics between groups. Although the area under the curve (AUC) of the normalized modularity (*Q*) was higher in liberals than conservatives at the trend level, there was no significant group difference in the network metrics when the correction for multiple comparisons was applied.

Although we observed that liberals (*M* = 0.77, *SD* = 0.08) had higher normalized modularity (*Q*) of the brain network than conservatives (*M* = 0.73, *SD* = 0.07) in the trend-level of uncorrected results (*p* = 0.079), there was no significant group difference in the network metrics when after correction for multiple comparisons (Fig. 2).

### Associations between intrinsic functional organization and RQT measures

A stronger anticorrelation between the left OFC and the precuneus was inversely correlated with the greater RQT total (*ρ* = − 0.50, *p* = 0.050) and self-regulation subscale scores (*ρ* = − 0.59, *p* = 0.017) in conservatives. Among the RQT self-regulation subscales, the causal reasoning and impulse control scores were also correlated with the connectivity between the left OFC and the precuneus in conservatives (*ρ* = − 0.60, *p* = 0.015; *ρ* = − 0.51, *p* = 0.045). At the trend level, there was a correlation between the RQT self-regulation subscale score and right insular connectivity with the left frontal pole/OFC (*ρ* = − 0.46, *p* = 0.073). None of the intrinsic functional organization measures were associated with RQT scores in liberals.

## Discussion

To expand the neurobiological understanding of different psychological responses to stress in liberals and conservatives, we investigated associations between intrinsic brain organization and psychological resources, such as resilience and self-regulation capacity. We found greater psychological resilience and self-regulation capacity in conservatives than liberals and moderates. We also found that greater impulse control and causal reasoning contributed to the regulation capacity in conservatives. Conservatives had stronger intrinsic connectivities between the OFC and the precuneus and between the insula and the frontal pole/OFC than liberals, and these brain organization characteristics are associated with the buffering roles of psychological resources. These results identified the neural underpinnings that may help conservatives achieve greater psychological well-being and life satisfaction despite the susceptibility to psychological stress caused by threats and anxiety.

Political psychology studies have investigated liberals’ and conservatives’ psychological responses to politically relevant social events and the accompanying stress because political perspectives are interactively influenced by threats and cognitive conflict perceived from social environments^4,7^. Regardless of political attitudes, people respond sensitively to stressful events that occur in a politically relevant social environment^3^. However, liberals and conservatives differ in the ways in which they recognize and respond to various political and social issues. Political psychologists have explained these psychological differences on the political spectrum from the perspectives of morality. Though relationships between ideology and morality is not simply explained in terms of a causal model, liberals are more likely to prioritize some moral values like care/harm, liberty/oppression, and fairness/cheating, whereas conservatives are more sensitive to a broader range of moral issues^31–33^. In addition to the different moral intuitions, they are thought to have different psychological resources and neurocognitive strategies. Liberals use a neurocognitive strategy that facilitates complementary cognitive control when facing conflicting information^10^, and this resource may allow them to accept more politically liberal views. However, it is still unknown which psychological resources and neurocognitive strategies allow conservatives to cope with the stress resulting from the sensitive perception of threatening and anxious situations while still having no physiological reactions to those stimuli and achieving enhanced satisfaction with their lives^15–17^. Compared to liberals, conservatives are more likely to conform to social rules and control their internal and external environments^19,22^. Along with a greater self-regulation capacity in conservatives, we found that they are more resilient to psychological stress. As psychological resilience and self-regulation lessen stress and enhance psychological well-being^21,34^, we suggest that these psychological resources of conservatives help them cope with the stress in a self-protective manner from threatening and anxious social circumstances and achieve greater life satisfaction.

Among various factors affecting psychological resilience^35^, we identified self-regulation capacity as the psychological resource that might be used to manage stress and improve psychological well-being in conservatives, while sociability (empathy and reaching out) and positivity (optimism and self-efficacy) were not as influential as the regulation capacity. We then showed that the greater self-regulation capacity was a consequence of a higher degree of impulse control and causal reasoning in conservatives. These psychological traits may be related to their personalities. The most evident personality trait of conservatives compared to liberals is conscientiousness^36^. Conscientiousness consists of low-order structures, such as orderliness and impulse control^37^; thus, we assume that conservatives, based on this personality trait, are more likely to follow orders and control the impulsiveness that possibly causes problematic outcomes. Having the tendency towards impulse control is also linked to affective well-being and life satisfaction^34^. Likewise, identifying problems and understanding causal relationships are strategies for managing psychological stress, and the causal reasoning may further improve psychological well-being^35,38^. Our findings provide evidence that greater impulse control and causal reasoning contribute to the psychological buffering roles of both resilience and self-regulation capacity in conservatives.

Neuropolitics research has examined the self-regulatory processes of single brain regions^10,14,39,40^; however, these limited regions are not sufficient to explain the complex structures of the psychological responses that manage stress^24^. Thus, we adopted intrinsic brain organization analyses to investigate multiple regions communicating with each other and to expand the neurobiological understanding of neuropolitics^25^. Conservatives had stronger rsFCs from the ACC, OFC, and insula that function cognitive and emotional processing to multiple brain regions of the default mode network (DMN), frontoparietal network (FPN), and dorsal attention network (DAN) when compared with liberals. If the OFC and precuneus became more strongly connected, conservatives were more likely to be resilient to psychological stress and have greater self-regulation capacity, specifically impulse control and causal reasoning. The OFC is one of key regions involved in self-regulation and psychological resilience, while the precuneus processes emotion regulation as coactivated with the amygdala^24,41,42^. The connectivity between these regions is related to the self-processing of negative emotional distractors and mental representation^29^. Indeed, a disruption of the functional coupling between these regions explains dysfunctional emotional processing and the negative self-view of patients with major depression^29,43^. Together with the above evidence, we suggest that the stronger intrinsic connectivity between the OFC and precuneus is a neurobiological resource that makes conservatives resilient to psychological stress.

The greater self-regulation capacity of conservatives was also related to the stronger connectivity of the insula with the frontal pole/OFC, which was identified as a subregion of the DMN^44^. The insula, together with the dorsal ACC, forms a salience network (SN) that functions to detect the saliency of external stimuli and facilitates task-related processing by switching between the DMN and the central executive network^45,46^. Having an interaction with the DMN, the insula in the SN engages in an attentionally demanding task and cognitive control, especially attention shifting^47,48^. The attentional switching away from emotional stimuli is a primary strategy to regulate negative emotions and might explain the approach-avoidance motivational responses of conservatives^49,50^. Indeed, conservatives use an avoidance-based inhibitory strategy to prevent negative feelings and outcomes in the face of stressful events^50^. The association of insular connectivity with self-regulation capacity in conservatives is supported by a recent finding showing weaker insular connectivity with the frontal pole/OFC as a brain dysfunction in disruptive behavior disorders that are characterized by cognitive and emotional dysregulations^30^. Thus, we propose that the stronger connectivity between the insula and frontal pole/OFC in conservatives is the other neurobiological resource that enhances self-regulation and lessens psychological stress.

Even through our hypothesis-driven tests assess the neural associations with psychological resilience and self-regulation, we missed to provide psychological explanations for the rest of the intrinsic brain organization differences; however, we think that these neurobiological differences might reflect some other cognitive psychological characteristics of liberals and conservatives. Conservatives had stronger connectivity between the ACC and the SFG/dlPFC and between the insula and the occipital regions than liberals. The ACC plays a substantial role in conflict monitoring and interacts with the dlPFC in compensatory processes of top-down cognitive control^51^. Thus, the different connectivity between the ACC and the SFG/dlPFC may be related to the greater sensitivity to and simultaneously greater tolerance of conflicting information in liberals. On the other hand, we propose that the different insular connectivity with the occipital regions might reflect different personality traits related to novelty seeking behavior between liberals and conservatives^52^. The insula, especially the posterior subdivision, is associated with risk prediction during decision-making and the novelty-seeking personality trait^53^. In addition, an insular network with posterior brain regions, including the occipital cortex, is involved in monitoring environmental information, which may be involved in interest in new experiences^54^. Based on the linkage between the insula and the novelty-seeking trait, our finding of insular connectivity differences raises the question of the underlying neurobiological basis of greater openness in liberals, which should be addressed in future studies. In addition to the seed-based intrinsic connectivities, the segregation and integration patterns of the intrinsic global brain network were comparable between political attitudes. This might indicate that liberals and conservatives have different intrinsic brain organization structures confined to specific connections between regions involved in self-regulatory processes and personality traits, such as conscientiousness and openness to new experiences.

The present study has several limitations that should be addressed in future studies. First, the number of conservative subjects was relatively small for high statistical power. We recruited a sufficient number of study participants at first, but most participants had moderately liberal or moderate attitudes. This might reflect the nature of political attitude distribution, which is skewed towards moderately liberal and moderate among the young adult population in the Republic of Korea^55^. The small sample size of conservatives may have increased risk of false discovery^56^; however, the results of intrinsic connectivity differences were replicated in the regression analysis that employed the large enough samples. Thus, our findings suggest the association between intrinsic connectivities and political attitudes are likely to be true positives. Second, extreme partisan attitudes, regardless of political orientation, might have influenced our behavioral results because there were individuals with strong political attitudes (i.e., “very liberal” or “very conservative”) only among the liberals. A recent study has shown the effects of partisan extremity on mental rigidity^57^. However, we argue that our participants with a very liberal orientation may not be so radical as to have extremely high mental rigidity because we observed comparable psychological resilience and self-regulation capacity between liberals and moderates. Third, the sample characteristics could be biased because the present study involved only young adult participants. However, our findings are in line with previous neuropolitics studies with populations with similar sociodemographic characteristics (e.g., college students or young adults, distribution of political attitudes, socioeconomic status)^10,13,40^. However, replicate studies with large samples across a broad age range are still needed. Finally, we assessed only one axis of political attitudes (liberalism vs. conservativism) even though other axes (economic freedom, government control, etc.) could exist in diverse socioeconomic and cultural situations in the real world^58^. However, the simple questionnaire we used to ask a degree of political attitude between liberal and conservative beliefs has been validated as a reliable measure of political attitudes in many other studies of political psychology and neuropolitics^10,13,32,52^.

In conclusion, we identified that conservatives, compared to liberals and moderates, have greater psychological resilience and self-regulation capacity that may buffer the stress caused by threatening and anxiety-inducing political information. These psychological resources are neurobiologically related to the stronger connectivities of the OFC and insula with the precuneus and frontal pole/OFC, respectively, in the intrinsic brain network of conservatives. This study may provide evidence explaining the different responses of liberals and conservatives to politically relevant social issues and expand the neurobiological understanding of neuropolitics by showing the interactions of brain regions that are involved in self-regulatory processes and resilience.

## Methods

### Participants

One hundred sixteen healthy volunteers were recruited from the student participant pool of the Transdisciplinary Research Center for Culture-Brain Dynamics at Seoul National University (SNU). We collected sociodemographic information, including political attitudes and socioeconomic status^59^. Political attitude was asked using a five-point Likert scale measuring the current political orientation ranging from very liberal (1) to somewhat liberal (2), moderate (3), somewhat conservative (4), and very conservative (5)^13^. We assigned participants to liberal (1, 2), moderate (3), and conservative groups (4, 5). To ensure an absence of mental illness, we conducted an interview using the Structured Clinical Interview for DSM-IV Axis-I Disorders, Non-Patient edition^60^. Additionally, we administered the Beck Depression Inventory (BDI) and Beck Anxiety Inventory (BAI) to assess the severity of depression and anxiety symptoms^61,62^. We excluded participants who met the following criteria: a lifetime history of psychiatric/neurological disorders, severe depression/anxiety symptoms, or exposure to psychiatric medications. After this screening process, one hundred two (42 liberals, 44 moderates, and 16 conservatives) of the original 116 participants remained (Table 1). We assessed psychological resilience and self-regulation capacity using the Korean version of the RQT, which consists of a total score for resilience and three subscores for self-regulation, sociability, and positivity^35,63^. All participants were informed of a complete description about all experimental procedures, and we were provided with written informed consent obtained from the participants. All experimental protocols and methods in this study were approved by the Institutional Review Board at SNU and performed in accordance with ethical guidelines of the Declaration of Helsinki.

### Image acquisition and preprocessing

We acquired functional and structural brain images using a 3 T Trio magnetic resonance imaging (MRI) scanner (Siemens Magnetom Trio) with a 32-channel head coil at SNU. Functional imaging data were acquired using a gradient echo-planar imaging pulse sequence (echo time = 30 ms, repetition time = 2000 ms, flip angle = 80°, voxel size = 3.4 × 3.4 × 3.4 mm^3^, and 34 slices). We instructed participants to relax with their eyes open during the session. Subjects were reminded to stay awake before the acquisition and monitored through the use of an eye tracker to ensure that they did not fall asleep. High-resolution T1-weighted brain imaging data were also collected using a magnetization-prepared rapid gradient echo sequence with the following parameters: echo time = 2.19 ms, repetition time = 2400 ms, flip angle = 8°, voxel size = 0.85 × 0.85 × 0.85 mm^3^, 192 slices).

We preprocessed the brain images using the CONN toolbox v17a^64^, implemented in the Statistical Parametric Mapping toolbox version 12 (SPM12; https://www.fil.ion.ucl.ac.uk/spm/), with the following pipeline. After discarding the first four volumes for gradient field stabilization, the functional brain images were corrected for slice-timing discrepancies and realigned to the first scan via rigid-body alignment^65,66^. Subjects who exhibited head movement exceeding the criteria (translation > 1.5 mm and rotation > 1.5 degree in any direction) were excluded. After reorienting a center point of the images at the anterior commissure, we coregistered the functional to structural images. Using a nonlinear warping algorithm, both images were subsequently normalized to Montreal Neurological Institute (MNI) space and resampled to 2 mm isotropic voxel dimension^67^. The functional images were spatially smoothed with a full width at half maximum Gaussian kernel of 4 mm to increase the signal-to-noise ratio^68^. We cleaned noise signals by conducting nuisance regression using a component-based noise correction method (CompCor), linear detrending, and temporal bandpass filtering (0.008 < *f* < 0.09 Hz)^69,70^.

### Functional connectivity analysis

To investigate seed-based functional connectivities, we selected the regions of interest (ROIs) according to previous neuroimaging studies reporting brain function in self-regulation and psychological resilience as follows: the ACC, amygdala, insula, and OFC^10,14,24,39^. The ROIs were defined using the Harvard–Oxford cortical and subcortical atlases. We calculated the connectivity strength using Pearson’s bivariate correlation between the seed regions and voxels in the rest of the brain from the individuals’ functional imaging data^64^. Subsequently, we transformed the correlation coefficients to a normal distribution by Fisher's *z* transformation. In addition to the functional analysis, we additionally conducted volumetric analyses using structural brain images for a replication study of previously reported neuroanatomical correlates in neuropolitics (Supplementary Methods & Results)^13^.

### Graph theoretical analysis

We also conducted graph theory analysis to investigate a whole-brain topographical brain network complementing the functional connectivity analysis using the selected ROIs. A connectivity matrix for each participant was computed using bivariate correlation between each pair of 106 ROIs, followed by the *r*-to-*z* transformation. Single-subject connectivity matrices were binarized after thresholding by the proportion of the strongest connectivity strength^71^. To avoid a selection bias for one specific threshold, we applied a wide range of the sparsity threshold (0.03 ≤ *S* ≤ 0.35) and calculated the area under the curve (AUC) for each network metric^72^. Using the Brain Connectivity Toolbox, we assessed the segregation and integration of the global brain networks by clustering coefficients, modularity, and global efficiency^73^. All the network metrics were normalized by comparing them to 1000 random networks, which preserved the size, density, and degree distribution of the original networks^72^.

### Statistical analyses

Because our samples are unequally sized, we checked the homogeneity of variance assumption before testing sociodemographic and psychological differences. The Fligner–Killeen test showed unequal variance of the RQT sociability variable; all the other variables were homoscedastic^74^. Sociodemographic variables were compared among liberals, moderates, and conservatives using the chi-squared test or one-way analysis of variance (ANOVA). To address the issue of unbalanced samples in terms of Type I error, we performed a MANOVA with Pillai’s trace method when comparing the RQT total and subtotal scores between groups^75^. As self-regulation, in addition to resilience, is assumed to be the other psychological buffer, we conducted an additional MANOVA to compare the RQT self-regulation subscales (emotion regulation, causal reasoning, and impulse control) among groups. The above analyses were followed by the univariate Welch’s ANOVA with Games-Howell post hoc comparisons, which are robust in case of unbalanced or heteroscedastic data, in SPSS version 23 (IBM, Armonk, N.Y.)^76,77^.

We compared the connectivity strength between 42 liberals and 16 conservatives using two-tailed independent samples *t*-tests. Additionally, we conducted a linear regression analysis with the political orientation score as a predictor of resting-state brain connectivity in total one hundred two participants. This was to demonstrate the validity of the group comparison analysis with the small samples of conservatives, which was potentially vulnerable to false discovery rate. We defined regions of significant difference if the clusters survived the voxel-level height threshold with an uncorrected *p* < 0.001 and cluster-level extent threshold of the false-discovery rate (FDR) with a corrected *p* < 0.05 to address multiple comparison issues^78^. We examined the homoscedasticity of the clusters detected by using the Fligner–Killeen test. For group comparisons of the network metrics, we performed nonparametric permutation tests with 10,000 repetitions, followed by the Bonferroni correction for multiple comparisons^79^.

To explore the neural associations with resilience and self-regulation capacity, we conducted Spearman’s correlation between intrinsic brain organization measures and the RQT scores within each group.

## Supplementary information

Supplementary file1
