# Supplementary material for: Intrinsic functional connectivity of blue and red brains: neurobiological evidence of different stress resilience between political attitudes
Source: Sci Rep. 2020 Sep 28;10:15877. doi: 10.1038/s41598-020-72980-x (PMC7522714; doi:10.1038/s41598-020-72980-x)
Supplement: Supplementary file 1 — Supplementary file1 [file 41598_2020_72980_MOESM1_ESM.docx]

Supplementary document

**Intrinsic Functional Connectivity of Blue and Red Brains: Neurobiological Evidence of Different Stress Resilience between Political Attitudes**

Taekwan Kim^1^, Ji-Won Hur^2^, Seoyeon Kwak^1^, Dayk Jang^3^, Sang-Hun Lee^1^, Jun Soo Kwon^1,4,5*^

^1^Department of Brain and Cognitive Sciences, Seoul National University College of Natural Sciences, Seoul, Republic of Korea

^2^Department of Psychology, Korea University, Seoul, Republic of Korea

^3^Interdisciplinary Program in Cognitive Science, Seoul National University College of Liberal Studies, Seoul, Republic of Korea

^4^Department of Psychiatry, Seoul National University College of Medicine, Seoul, Republic of Korea

^5^Institute of Human Behavioral Medicine, SNU-MRC, Seoul, Republic of Korea

Supplementary Methods

We preprocessed the T1-weighted images in the Statistical Parametric Mapping toolbox version 12 (SPM12; http://www.fil.ion.ucl.ac.uk/spm/). We segmented the images into grey matter, white matter and cerebrospinal fluid using the unified segmentation algorithm. We registered the structural brain images to a study-specific template using a high-dimensional Dartel algorithm and resliced them into the Montreal Neurological Institute space^1^. The gray matter images were modulated and spatially smoothed with a full width at half maximum Gaussian kernel of 8 mm.

The mean gray matter volumes were extracted from ROIs (ACC and bilateral amygdala) to see if the previous findings would be replicated in our samples of the Asian population^2^. We conducted both ROI-based regression and whole-brain voxel-based morphometry (VBM) analyses to examine neuroanatomical correlates with political orientation score using one hundred two participants. We included age, sex, and total intracranial volume as covariates to control for confounding effects on associations between brain structures and political attitudes. We defined regions showing significant correlations (1) if a cluster-level corrected false-positive detection rate of *p* < 0.05 with a peak-level threshold of *p* < 0.001 and a cluster size (k) larger than 50 in the VBM analysis; (2) if p < 0.016 (corrected for the numbers of selected brain region) in ROI analysis.

Supplementary Results

We observed no brain structural associates with political orientation score when multiple comparison corrections were applied to the VBM results. Under a more lenient threshold of uncorrected *p* < 0.001, we found that conservative attitude was associated with larger superior occipital gyrus (peak at 16, -94, 26; *T* = 4.02; *k* = 88) and precentral gyrus (peak at 46, 0, 36; *T* = 3.56; *k* = 55) in right hemisphere. In addition, the ROI analysis showed that the ACC (*β* = -0.16, *p* = 0.31), right amygdala (*β* = 0.15, *p* = 0.35), and left amygdala (*β* = -0.08, *p* = 0.63) did not significantly predicted political orientation score. Like the study by Kanai et al. (2011), we did not observe any significant results in the whole-brain analysis; however, neural correlates of amygdala and ACC with conservativism and liberalism, respectively, were not replicated in the ROI analysis as well.

Table S1. Intrinsic functional brain connectivity associated with political orientation score in the regression analysis

| Seed region | Brain region | MNI coordinates (mm) | Cluster size (*k*) | Statistics | |
| --- | --- | --- | --- | --- | --- |
|  |  |  |  | *T* | Cluster-extent *p*^a^ |
| *Predictor: political orientation score* | | | | | |
| ACC | L SFG/dlPFC | -20, 14, 62 | 72 | -4.17 | 0.016 |
| R OFC | Precuneus | -8, -54, 50 | 395 | -5.04 | < 0.001 |
| L insula | L occipital pole | -2, -92, 22 | 134 | 5.37 | < 0.001 |

References

1. Ashburner, J. A fast diffeomorphic image registration algorithm. *Neuroimage.* **38,** 95-113 (2007).

2. Kanai, R., Feilden, T., Firth, C. & Rees, G. Political orientations are correlated with brain structure in young adults. *Curr Biol.* **21,** 677-680 (2011).
